# Interviews with experts in rare diseases for the development of clinical decision support system software - a qualitative study

**DOI:** 10.1186/s12911-020-01254-3

**Published:** 2020-09-16

**Authors:** Jannik Schaaf, Hans-Ulrich Prokosch, Martin Boeker, Johanna Schaefer, Jessica Vasseur, Holger Storf, Martin Sedlmayr

**Affiliations:** 1 Medical Informatics Group (MIG), University Hospital Frankfurt, Frankfurt, Germany; 2 Chair of Medical Informatics, Department of Medical Informatics, Biometrics and Epidemiology, Friedrich-Alexander University Erlangen-Nürnberg, Erlangen, Germany; 3 Institute of Medical Biometry and Statistics, Medical Faculty and Medical Centre – University of Freiburg, Freiburg, Germany; 4 Institute for Medical Informatics and Biometry, Carl Gustav Carus Faculty of Medicine Technical University of Dresden, Dresden, Germany

**Keywords:** Rare diseases, Clinical decision support systems, Computer-assisted diagnosis, Qualitative research, Interview

## Abstract

**Background:**

Patients with rare diseases (RDs) are often diagnosed too late or not at all. Clinical decision support systems (CDSSs) could support the diagnosis in RDs. The MIRACUM (Medical Informatics in Research and Medicine) consortium, which is one of four funded consortia in the German Medical Informatics Initiative, will develop a CDSS for RDs based on distributed clinical data from ten university hospitals. This qualitative study aims to investigate (1) the relevant organizational conditions for the operation of a CDSS for RDs when diagnose patients (e.g. the diagnosis workflow), (2) which data is necessary for decision support, and (3) the appropriate user group for such a CDSS.

**Methods:**

Interviews were carried out with RDs experts. Participants were recruited from staff physicians at the Rare Disease Centers (RDCs) at the MIRACUM locations, which offer diagnosis and treatment of RDs.

An interview guide was developed with a category-guided deductive approach. The interviews were recorded on an audio device and then transcribed into written form. We continued data collection until all interviews were completed. Afterwards, data analysis was performed using Mayring’s qualitative content analysis approach.

**Results:**

A total of seven experts were included in the study. The results show that medical center guides and physicians from RDC B-centers (with a focus on different RDs) are involved in the diagnostic process. Furthermore, interdisciplinary case discussions between physicians are conducted.

The experts explained that RDs exist which cannot be fully differentiated, but rather described only by their overall symptoms or findings: diagnosis is dependent on the disease or disease group. At the end of the diagnostic process, most centers prepare a summary of the patient case. Furthermore, the experts considered both physicians and experts from the B-centers to be potential users of a CDSS. The experts also have different experiences with CDSS for RDs.

**Conclusions:**

This qualitative study is a first step towards establishing the requirements for the development of a CDSS for RDs. Further research is necessary to create solutions by also including the experts on RDs.

## Background

According to the World Health Organization (WHO), a disease is defined as rare if it affects up to 1.3 of every 2000 individuals [1]. While studies have proven that significant discrepancies or wrong diagnoses occur in 10–20% of diseases, the situation in rare diseases (RDs) is even worse [2].

RDs are often chronic and degenerative, affecting multiple organ systems and often causing unclear symptoms [3]. It is estimated that about 5000 to 7000 different RDs exist. Overall, the identification of these diseases is a challenge for physicians [4]. A study in Australia showed that 30% of patients had waited for five or more years for a correct diagnosis. Some of these patients consulted more than six physicians before a correct diagnosis was made [5]. Geographical dispersion both of patients and medical RDs experts impedes the diagnosis of RDs. Furthermore, limited or inconsistent studies, few medical experts, few patient cases and incomplete information are available, which limits the amount of data for research and care [6].

The MIRACUM consortium (Medical Informatics in Research and Medicine), which is funded by the German Ministry of Education and Research (BMBF), is a large research network which includes 10 university hospitals. The consortium aims to create data integration centers (DICs) at each location and to make data available through interoperable technologies for research and patient care [7]. The linking of datasets in this large research network provides an opportunity for research and diagnostic support regarding RDs.

The benefit of data sharing between the university hospitals in the MIRACUM consortium will be demonstrated by different use cases. One of the use cases is the conception and development of a clinical decision support system (CDSS) for RDs, which will be called DISERDIS (Diagnosis Support in Rare Diseases). This CDSS aims to identify similar patients to an undiagnosed patient in the DIC, which could give a physician an hint for diagnosis [8].

Sim et al. defines a CDSS as a software system in which patient’s characteristics are matched to a knowledge base and recommendations or assessments for clinical decision making are presented to the physician. A knowledge base is a collection of data which provides the necessary information to make clinical decisions [9]. CDSS can have various application areas such as medication safety [10], infection control [11] and diagnostic support [12]. In this publication, we use the term CDSS in the context of diagnostic support.

During the development of a CDSS, the users of the system should be involved in every phase of development to increase efficiency and user satisfaction [9, 13–15]. In our case, the users work at the Rare Diseases Centers (RDCs) within the participating hospitals. These centers serve as specialized institutions in the hospitals for patients without a diagnosis. RDCs are divided into A, B and C-centers [16]. A-centers and B-centers are part of a hospital. The type A-center includes a center-guide, who is typically a physician with a qualification in a specialty, who guides the patient to the right place in the healthcare system to get a correct diagnosis. Therefore, they act as a reference center. A-centers include more than two B-centers (center of expertise), which offer outpatient and inpatient care for a specific RD or RDs groups (e.g. center for rare lung diseases). Their task is to treat or confirm suspected diagnosis of patients with a specific RD. B-centers can be part of a department in a hospital. For instance, the university hospital Frankfurt includes a reference center (A-center) for RDs. This center includes different B-centers, e.g. a center for cystic fibrosis which is part of the department of pulmonology. C-centers are located outside a hospital and provide outpatient care for certain RDs [17].

A User-Centered Design Process (UCD) defines a problem-solving process in a multistage way, in which user requirements, needs and limitations are investigated and prototypes are designed, developed and tested with the users [18]. When developing a software system for healthcare, the acceptance of the software depends on successful integration into clinical processes and organizational conditions [19]. In a systematic review of CDSS, Kawamoto showed that it is necessary to provide decision support as part of the physician’s workflow and to deliver the decision support at the right time and location [20]. Furthermore, in accordance with our above definition of a CDSS, it is important to investigate which clinical data in the knowledge base should be used for decision support. Additionally, human factors play a significant role in the usability and acceptance of the software. Therefore, it is important to ensure the CDSS is used by the appropriate user group [21].

In the past, several CDSSs for RDs have been developed and published [22–25]. Most of these studies only focused on the performance of the CDSS and the accuracy of the diagnosis. To our knowledge, there is no study available which investigates the user requirements and needs for a CDSS in RDs. Therefore, we conducted this study as starting point of a UCD in the development of CDSS software that can be adapted to the characteristics and needs of the user [26].

The objectives of this study are to investigate (1) the relevant organizational conditions for the operation of a CDSS for RDs (e.g. the diagnostic workflow), (2) which data is necessary for decision support, and (3) the appropriate user group for such a CDSS.

## Methods

### Design

This qualitative study with expert interviews was performed in the context of the MIRACUM consortium to gather requirements for the development of CDSS software for RDs. As part of our UCD, this qualitative design was chosen because it enabled us to obtain relevant information, insights, and the preferences and needs of all stakeholders [27].

To enable trustworthiness in our qualitative study, we conducted the study with the concept-driven (deductive) approach of Mayring. The study was also performed in accordance with the Consolidated Criteria for Reporting Qualitative Research (COREQ) [28, 29]. We provide a checklist for COREQ and 31 out of the 32 items of COREQ were considered in this study (see Additional file 1). To enable data accuracy in data collection, the transcripts were checked and approved by the participants. Furthermore, results of data analysis were discussed between two authors and presented to the participants. The methods, described in the next sections in more detail, were chosen to fit a logical, precise and traceable research process to yield meaningful and useful results.

### Setting and sampling

For this study, purposeful sampling was used [27, 30] in which experts in RDs, known by the authors, were invited to participate in the study. With this method, we tried to maximize the variation in expert competencies and took the following characteristics of study participants into account: type of medical center where specific RDs are diagnosed and treated (RDC), member of the MIRACUM consortium, completed medical degree and completed specialist qualification in human medicine. Based on these criteria, we identified eight potential study participants, since 8 of 10 hospitals in MIRACUM have established an RDC. The participants were familiar with the objectives and background of the study. In the context of this study, an expert is defined in accordance with Meuser and Nagel as a person who has knowledge in a research context that is not accessible to everyone in the field of expertise. They can act based on their experience and knowledge [31].

For recruitment, the participants were contacted by email. If there was no reply to the email within two weeks, the experts were contacted by telephone.

### Instrument

**Fig. 1:**
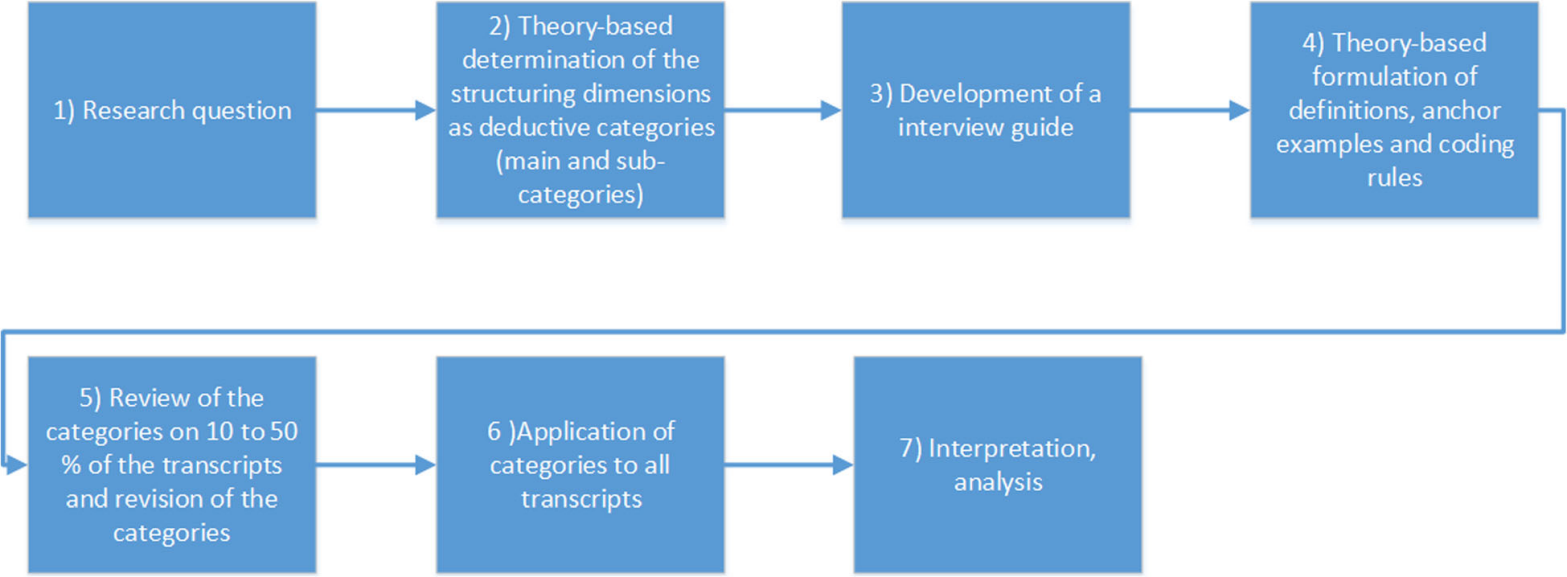
From research questions to analysis according to Mayring [32]

An interview guide with eight questions was prepared in German by JAS reflecting the research objectives (see Additional file 2). For this qualitative study, we followed the concept-driven (deductive) approach of Mayring [32] with 7 steps as shown in Fig. 1.

The development of the interview guide follows an approach suggested by Helfferich and Kuckartz [33, 34]. Key terms from the research questions were derived prior to defining deductive categories [32]. We used a pragmatic approach to define the key terms involving an expert in RD at each institution (shown in Table 1) and no theory or model was considered. First, key terms were collected. We then checked the terms for already-known aspects in these terms. All terms that only included prior knowledge were deleted. Lastly, key terms were sorted and assigned to our research questions.

**Table 1:** Key terms derived from research questions

Research question	Key terms
Which organizational conditions are relevant for the operation of a CDSS for RD?	•Admission to the center •Steps before and after a definition of a diagnosis •Persons involved in the diagnostic process •Usage of patient findings
Which data is necessary for the decision support of RDs?	•Clinical characteristics of patients with RD •Patient findings •Which findings are particularly relevant? •Current patient documentation •Collection of patient information
What is the appropriate user group for a CDSS for RDs?	•Usage of current CDSS •Users of a CDSS

Based on the key terms, deductive categories divided into main- and sub-categories (shown in Additional file 3 - Part A) were defined (Fig. 1, step 2). We then created an interview guide containing the questions the interviewed experts were asked (Fig. 1, step 3). For the purpose of this publication, the interview guide was translated from German to English. The questions were designed as open questions. Furthermore, control questions were included to confirm what had been said so far, as well as questions to maintain the flow of the conversation [35]. The first version of the interview guide was pretested by JAS with an expert on RDs and revised with only minor changes.

### Data collection

Participants were interviewed face-to-face in their own offices. No further persons were present. Interviews were conducted once and not repeated. Due to time scheduling issues for one of the participant, one interview was conducted by telephone.

The interviews were conducted from June to September 2019 in the German language. The length of the interviews ranged from 25 to 60 min. At the beginning of the interviews, participants were asked to answer a short questionnaire. All interviews were conducted by JAS, whose researcher characteristics are the following: “gender: male”, “experience: three years research experience in medical informatics”, “degree: M.Sc. in Medical Informatics” and “occupation: Research assistant and PhD student”.

Data collection continued until all interviews were finalized. Saturation was reached by the time (1) all participants successfully completed the interviews and (2) categories were adequately represented in the data after the refinement of the categories, whereas the final category system could be applied to the whole data [36].

### Data analysis and processing

The interviews were recorded on an audio device and then transcribed into written form by JAS using Microsoft Office Word 2016; data management was also performed with this software. Transcripts were checked by MS for validity, and then returned to the participants for validation. All participants approved the transcripts.

The transcription was performed according to the following rules of the transcription system of Kuckartz: participants were anonymized, i.e. all identifying data were removed from the transcripts. The actual transcription was literal, ignoring dialects, non-verbal expressions or special emphases and slightly smoothing the language [35].

Before applying qualitative content analysis as described by Mayring, anchor examples and coding rules were defined (Fig. 1, step 4) [32]. Anchor examples serve to show which text passages can be assigned to each category. Coding rules describe when a text passage can be assigned to a category.

As recommended, 10–50% of the transcribed material was checked in advance using the category system to determine whether the categories were adequately represented in the data (shown in Additional file 3 – Part A) [33]. To this end, two (*n* = 2) interviews were selected (Fig. 1, step 5). Some categories could not be assigned to text passages or were not represented in the data. Categories were therefore re-grouped or removed (described in Additional file 3 – Part B). Finally, a revised category system with nine categories was created (shown in Additional file 3 – Part B and C). Following revision, the category system was applied to the entire data material (Fig. 1, step 6) [32]. For quality assurance of accuracy and authenticity, the recordings were checked and replayed once [37].

All transcripts were read and analyzed by JAS (Fig. 1, step 7). Whenever a text passage could not be directly assigned to a category, the category chosen was discussed and decided by JAS and MS Results in each of the categories were presented and discussed by the participants and all authors in an online video conference. All participants approved the findings.

After assigning text passages to categories, all text passages within each category were summarized and selected passages were chosen to represent the content of each category.

To synthesize the results, quotations from the experts were used which best represented the content of a category. The quotations were translated from German to English.

## Results

### Participants

Seven out of eight experts responded to the invitation. One of the potential participants could not be reached by phone or email. All of the experts who responded to our invitation participated in the study. Therefore, we achieved a high participation rate of 87.5%. Characteristics of study participants are shown in Table 2.

**Table 2:** Characteristics of the study participants

Characteristics	Options	Participants (***n*** = 7)
Gender	female	2
	male	5
Age group	> 59	2
	50–59	2
	40–49	2
	30–39	1
Medical specialty	nephrology	1
	neurology	1
	immunology	1
	pediatrics	2
	internal medicine	1
	psychiatry and neurology	1
Years of experience in the field of rare diseases	30	1
	25	1
	24	1
	15	2
	4	2
Prior experience with clinical decision support systems	Yes	3
	No	4

The participants were predominantly male (*n* = 5) and the age range was wide. The study participants represented several different medical specialties: nephrology (*n* = 1), neurology (*n* = 1), immunology (*n* = 1), pediatrics (*n* = 2), internal medicine (*n* = 1) and a double qualification (neurology and psychiatry). Their experience with RDs ranged from 4 to 30 years, with an average of 16.7 years. Three of the seven study participants had prior experience with CDSSs.

### Main themes by deductive category

In the following sections, the results are presented and organized by research questions and deductive categories. References for selected quotations are provided for each statement (see Additional file 4). We also provide exemplary quotes in each paragraph, abbreviated by “Q” and numbered in ascending order (e.g. Q1).

#### Relevant organizational conditions

##### Steps before a consultation with a patient

When asked to define the steps before a consultation with a patient at the center, the experts explained that medical or administrative center guides receive documents from the patients (e.g. via mail) or directly from the treating physician, typically private practitioners or doctors from other hospitals (Q1-Q3). One expert explained:

“*The typical diagnostic route actually operates through our guides and coordinators, where the patients first report at the center and the center guides review the documents [ … ]. And then the patients are referred directly to a B-center, where the patients are then seen or actually first examined by the center guide.*” (Q1, translated from German)

One expert pointed out that patients referred from a university hospital are prioritized for a diagnosis (Q4).

After the center guides have inspected the documents, the patient case is assessed and a recommendation is made (Q5). One expert stated that an interdisciplinary case conference or discussion is part of the assessment of the patient case. In these conferences, physicians review and discuss cases together, and a decision is made as to whether or not the patient will be referred to a B-center (Q6).

#### Persons involved in the diagnostic process

The experts mentioned that administrative and medical center guides and various experts at the B-centers are involved in the diagnostic process (Q7–9). One expert stated:

“*Once a month we have a case conference where we try to solve these patient cases. Every B-center is involved in this.*” (Q8, translated from German)

Two experts gave examples of health professionals from different clinical specializations who are involved in the diagnostic process, for instance: neurology, pediatrics, general medicine, otorhinolaryngology, laboratory medicine, pulmonology and immunology (Q10–11, Q13–14).

One expert also pointed out that a specialist in psychosomatic is part of their team, because some cases are not of somatic origin (Q12). Four experts explained that they perform interdisciplinary case discussions in their centers in order to analyze the patient cases (Q8, Q15–17). One of these experts indicated that in his center, large case discussions are organized in a kind of lecture. The goal is to discuss difficult patient cases to obtain further opinions (Q18).

### Usage of clinical findings

Clinical findings are mainly used by the medical center guides, the experts at the B-centers, and also the specialists from the outpatient department (Q19–23). An expert stated:

“*Then the patients are referred directly to a B-center, where the patients are then viewed or actually first examined by the center guide.*” (Q19, translated from German)

Another explained:

“*Either we see quite clearly that it is a rare movement disorder and then we would forward the documents very quickly to the appropriate B-center. And it doesn’t cost the center guide most of their time, rather the cases with a bundle of symptoms take a lot of time, which also have been clarified intensively.*” (Q22, translated from German)

#### Clinical data for diagnosis of RDs

##### Clinical characteristics and findings of RDs

Regarding the relevant clinical features of RDs, two experts mentoined that characteristics of RDs do not differ from common diseases and that no general symptoms could be reported. The overall combination of symptoms is important when an unusual symptom or patient history is described (Q24–25). One expert pointed out:

“*Many of the characteristics of patients with rare diseases are common. It’s not that they all have rare symptoms, but they all have very common complaints.*” (Q24, translated from German)

Another expert stated:

“*It is certainly not possible to give generally valid symptoms, because any symptom that is present in a rare disease can also sometimes occur in a common disease. But it always depends on the overall constellation. [ … ].*” (Q25, translated from German)

One expert explained that clinical characteristics depended on the disease group. In immunology, for instance, the antibody constellation is important in connection with symptoms, whereas in movement disorders the actual movement disorder is essential in connection with genetics. The expert also pointed out that it would be difficult to apply a universal strategy because each disease group has a different blend of symptoms:

“*Yes. That depends on the disease group. [ … ]. In rare diseases I think it's difficult to find a universal strategy for all these diseases.*” (Q26, translated from German)

For instance, in some cases neurological or psychological findings are important, whereas in pediatrics the morphology of the patient is likely to be important (Q27). One expert explained that many characteristics could not be depicted and are more or less coincidental: “*Ultimately, many things cannot be depicted in this way, many things are more or less coincidental.*” (Q28, translated from German).

One expert suggested that a patient’s family tree is a hint for the diagnosis, and the phenotype increases the likelihood of the diagnosis of a certain disease (Q29–30). On the other hand, two experts mentioned that there are also diseases that are phenotypically very different, such as immunodeficiencies or psychiatric diseases (Q31). Mental disorders, for example, have high a phenotypic variability. One expert described a family in which different family members had each developed different mental disorders as a result of a RD (e.g. depression or schizophrenia) (Q32).

Additionally, one expert also regarded the patient’s own description of their symptoms to be important (Q33).

##### Relevance of findings

One expert explained that certain findings or measurements are required for the diagnosis of certain diseases, while the same findings are completely irrelevant for other diseases:

“*[ … ].The findings I need for this are completely different. For one of them I need a lactate value. And for another, I'm not interested in the lactate value at all.*” (Q34, translated from German)

For example, the laboratory and imaging data, clinical symptoms and genetics are of interest to internists:

“*As an internist, one naturally always likes to look at the laboratory values. Are there any abnormalities in the laboratory or any abnormalities in imaging? What symptoms does the patient report? These are the central pre-diagnostics. [ … ].*” (Q35, translated from German)

For another expert, the epidemiology of the patient, e.g. age, sex and origin, is also of interest. One expert indicated that the travel history is also relevant in order to exclude rare infectious diseases (Q36).

##### Patient documentation

Five experts explained that they prepare a summary letter containing the most important findings and examinations regarding the patient. Some centers send this information back to the patient (Q37–41). One expert stated:

“*We summarize. That’s what the center guides do. They compile a summary letter of what they saw there*.” (Q37, translated from German)

Another expert stated:

“*[ … ] we send the patients a doctor's letter. The letter tries to represent the essential findings structured [ … ] in terms of comprehensibility and arranged according to symptom groups. Then the letter is sent back.*” *(*Q38, translated from German*)*

One RDC uses a standardized questionnaire containing the findings and the results of the case conference (Q42). Another RDC also documents whether exome sequencing would be required (Q43). In addition, this center continuously documents suspicious diagnoses in their electronic health record system (EHR) (Q44–45).

One expert pointed out that the documentation is stored in a separate database and not in their EHR. Additionally, patients’ demographic data are also documented there (Q46–47).

Another expert explained that documentation of chronic immunodeficiencies is performed in more detail, including medication, demographic data, diagnoses and diagnosis criteria (Q48–49).

#### Appropriate user group for a CDSS for RDs

##### Use of software tools for diagnostic support

When asked whether software tools are used for diagnostic support in their centers, two experts mentioned using the internet and medical databases such as Medline, Online Mendelian Inheritance in Man (OMIM) and Orphanet (Q50–51). OMIM is a database which contains descriptions of human genes and their relationship to phenotypes [38]. Orphanet is an organization which provides a database including information about RDs [39]. Three experts did not specify which software tools are used (Q52–54). One expert stated:

“*We have never used FindZebra. But apart from that we try to use tools, although I cannot tell you exactly what my colleagues use.*” (Q52, translated from German)

One expert cited Phenomizer, ADA, Isabel Healthcare and FindZebra as software tools for diagnostic support in their center:

“*On the one hand there are the special tools, of course, like FindZebra or Phenomizer, but we also strongly refer to Isabel Healthcare for example, which is more general. We can also access ADA.”* (Q55, translated from German)

With Phenomizer it is possible to measure the similarity between phenotypes and genetic diseases. The software suggests a disease based on the entered phenotypes [40]. FindZebra is a search engine for RDs and which finds related articles for entered patient characteristics, querying selected databases such as Orphanet, Wikipedia or Medline [41]. Isabel Healthcare is a web-based diagnostic support system that provides a weighted list of differential diagnoses based on entered symptoms [22]. ADA DX is a software with the potential to recognize RDs in their early stages [25].

One expert mentioned that there are currently no satisfactory software solutions to support diagnosis (Q56).

##### Users of a clinical decision support system

The experts considered medical center guides as well as the specialists in B-centers to be the likely future users of DISERDIS (Q57–61). One expert explained:

“*Who? Definitely the doctors.*” (Q57, translated from German)

Another expert stated:

“*Well, at the first level definitely the center guide, but at the second level the specialists in the B-centers*.” (Q60, translated from German)

One expert cautioned that administrative center guides should not be considered as users because they don’t have the necessary medical qualification to evaluate the patients (Q62). Another expert pointed out that the use of the system could depend on the quality of the CDSS. If the system was accurate enough, the center guide could use it. However, if the system required additional expert knowledge, the expert in the B-center would be a more suitable user (Q63).

Another aspect mentioned was that DISERDIS should also be used outside the centers, elsewhere in the clinic (Q64). One expert also suggested that users should be divided into two groups: those who filled in data entry forms (e.g. coding professionals) and medical center guides who follow the recommendations of the CDSS (Q65).

## Discussion

This qualitative study is a first step towards establishing which topics and conditions are important for the development of a CDSS for RDs. In summary, the objectives of this study were to investigate which relevant organizational conditions for the operation of a CDSS for RDs are available. Furthermore, we investigated how data should be processed to enable diagnostic support and who are potential users of a CDSS for RDs.

### Similar organizational conditions

The results of the study show that organizational conditions in the RDCs are similar. Administrative or medical center guides receive documents from the patients or treating physicians to inspect the patient cases. After these inspection, the patient case is assessed and a recommendation is made. For instance, case conferences are conducted, where different experts from different medical specialties or B-centers are involved.

### General data sets for RDs are not possible

Regarding our research question of which clinical data is necessary for a CDSS in RDs, the experts provided very different opinions about which clinical features and findings are important. A generally valid listing and description of symptoms for a specific RD is difficult or impossible, since different combinations of symptoms can occur in different disease groups. In RDs, the overall combination of symptoms is important. Moreover, symptoms in RDs do not differ from common diseases. The experts therefore pointed out that it would be difficult to apply a universal strategy to describe symptoms of RDs.

This result suggests that our envisioned CDSS cannot be based on a simple dataset to generally describe RD patients. This is reinforced by the experts’ statements on which clinical findings are relevant for the diagnosis of RDs. The experts mentioned different types of clinical findings. However, their opinions might be influenced by the fact that they are often specialized in certain RDs, and thus may only provide information which is relevant to their field.

Overall, these results correspond to the known high heterogeneity of RDs [42–44]. As an example, the European Reference Networks (ERNs) are subdivided into 24 different reference networks on different disease groups, e.g. “European Reference Network on Rare Bone Disorders” or “European Reference Network on Rare Respiratory Diseases”. These ERNs were established in a European legal framework to improve research and care in rare or complex diseases or conditions [45]. In a future investigation, a disease-group related data set for usage in the CDSS could be considered.

With regard to the documentation of patient data, most of the experts explained that summaries are used to describe the most important findings. When considering the definition of a CDSS, the knowledge base is important. These summaries could become a part of the knowledge base of the planned CDSS. However, future investigation should determine which data are described here, what qualities they have and how they are structured. Only two experts stated where the data are stored. Additionally, similarities between these summaries should be investigated to check whether they are a possible source for the knowledge base of the CDSS. In most cases, summaries are available in written text form and thus as unstructured data. In these cases, methods such as Natural Language Processing (NLP) may be applied to generate structured data from unstructured data [46, 47].

The question also remains of whether patient cases forwarded to other institutions will be followed up. Otherwise, the final diagnosis and findings of patients are not available and therefore not usable for a knowledge base.

### Center guides and RD experts as potential users

As clearly stated in the literature, it is essential to provide decision support at the right time and location as part of the physician’s workflow [20]. The results show that two options for workflow integration are possible: involvement of the center guides, and experts in the B-centers, both of whom were also identified as the possible users of a CDSS. Additionally, a suggestion by one expert shows, that users can be divided into two groups: those who filled out data forms (e.g. coding professionals) or medical center guides. Additionally the expert mentioned the CDSS could also be used outside the RDC, elsewhere in the clinic. Therefore it is necessary to assess this aspect in further studies and to determine where the CDSS can be used most effectively. However, only physicians should use the CDSS since they have the necessary medical qualification to evaluate the patient case. Additionally, the system could be applied in interdisciplinary case discussions to present and discuss cases. This scenario was explained by one expert in the interview.

This is consistent with the experts’ statements that prior experience with software for diagnostic support is common. Experts have different experiences with software for diagnostic support, e.g. with Phenomizer, FindZebra, ADA or Isabel Healthcare.

A further study might include these experiences and consider them in the development of our CDSS.

### Limitations

The current study has several limitations. It was only possible to include seven experts on RDs. According to the study’s inclusion and exclusion criteria, only experts in RDs from the MIRACUM consortium were selected for the interviews and no further stakeholders were involved who might have had a different view on the topic. Therefore, our results are limited to the RDC in the MIRACUM consortium. However, purposive sampling is useful for pilot studies and common in qualitative research [48]. Although the sample size was small, the study represented a diverse group of physicians with a wide range of experience in different medical specialties. Therefore the results are suitable in the context of our UCD. Moreover, we identified other studies in the context of RDs with only few study participants [49–52]. Nevertheless, the results are preliminary and further interviews should be conducted with experts of RDCs to get more insights. While the experts were all from Germany, we believe that our findings are also valuable to other countries, especially those with similar RDCs.

As with any qualitative research, qualitative expert interviews are not intended to produce representative and generalizable results [30, 53], but rather to examine expert opinions, in this case on what is necessary for the development of a CDSS for RDs. The opinions of the included experts are therefore valuable to increase the acceptance of the planned CDSS. To obtain representative data, a quantitative study should be conducted including all 32 established RDCs in Germany.

Qualitative research itself is strongly dependent on the competence of the interviewers and evaluators. The interviews were performed and analyzed by only one author, but were approved by all authors. However, implementing a high methodological standard with COREQ helped us to minimize possible bias across the study. Qualitative research can also be criticized as not having a purely inductive approach when deductive categories are used. Therefore, the use of an inductive approach where categories are defined based on the transcripts, or combining an inductive and deductive approach, may have been more appropriate [54].

Despite these limitations, qualitative studies also have some advantages, e.g. their detail and the depth at which they can be performed. Interviews are not limited to predefined questions and allow the interviewer to clarify answers in more detail. This offers an advantage over quantitative studies, where closed questionnaires are used. In addition, expert experiences often produce more convincing and efficient data than quantitative studies [55]. On the other hand, study participants are occasionally reluctant to elaborate on topics which may disclose a possible gap in their knowledge [56].

Looking ahead, continuing qualitative studies such as focus groups will enable us to jointly define requirements in detail with the experts. For instance, the study could include how a patient similarity in RDs patients could be calculated. A further type of qualitative studies could be a diary study. Diary Studies invite an expert to keep a diary of their daily experiences. For example, an expert in RDs could document his daily work with patients in a diary and thus name the requirements for a CDSS from everyday practice [57]. Further input for the requirements for such a CDSS could also be provided by RDs patients and their families. For instance Babac et al. have indicated in their study that the integration of patients in the medical decision-making process is relevant [58].

Furthermore, we will develop a low and high fidelity prototype to assess the CDSS early together with the experts. The use of a low fidelity prototype allows the involvement of experts at an early stage of the development of the CDSS. Low-fidelity prototypes do not allow user interactions, but they can represent the basic structure of a software system and thus provide a basis for discussion. In the next step, a high-fidelity prototype will be used, which allows user interactions [59, 60]. Further qualitative studies can be used to evaluate the high-fidelity prototype. One possibility is a thinking aloud test, where the users of a software are asked to communicate their thoughts aloud, while interacting with the software. During the interaction, they indicate why they perform interaction and what their goal is [59]. A further step could be a “near live” clinical simulation. This test scenario differs from the thinking aloud test in that the study participants are in a prepared treatment room, similar to the clinical routine. The study participants are confronted with different case scenarios, whereby the patient cases are simulated by actors recorded on a video tape. The participants can start and stop the video at their own. As in the thinking aloud test, the computer screen is recorded on video and an audio recording is made [61].

Whereas conducting expert interviews is a good and first starting point to get insights and relevant requirements for such a CDSS, it is necessary to assess the feasibility for the CDSS for RDs through the inclusion of the above-mentioned studies.

## Conclusion

To our knowledge, this qualitative study is the first involving experts on RDs to inform the development of CDSS software for RDs. Through the answers to our research questions, we determined that RDCs show similar organizational conditions in their diagnostic processes. RDCs receive patient cases from the treating physicians and check the patient cases in advance. This check is performed by administrative or medical center guides. In order to reach a diagnosis, they also include experts in the B-centers and conduct interdisciplinary case discussions, where physicians from different medical specialties are involved.

Regarding the definition of clinical data which can be used for diagnostic support, the experts agree that RDs cannot be mapped to a simple general data set due to their heterogeneity. It is difficult to describe RDs with overall symptoms, since RDs do not differ from common diseases regarding the symptom description and can occur in different disease groups. However, patient summaries are available in the centers which structure the main findings of a patient case.

Furthermore, the study shows that a CDSS is likely to be used by both medical center guides and experts in the B-centers. Administrative center guides should not use the CDSS as they do not have sufficient medical qualification to evaluate patient cases.

Finally, the study shows that experts in RDs differ in their prior experience with CDSS, since different CDSS for RDs are used.

In summary, this study makes an important contribution to determining the first requirements for the development of a CDSS for RDs. Looking ahead, further interviews with experts for RDs should be conducted to get deeper insights into their work.

## Supplementary information

**Additional file 1.** COREQ checklist.

**Additional file 2.** Interview guide.

**Additional file 3.** Category system for content analysis.

**Additional file 4.** Example quotations from the interviews.

## Data Availability

The datasets used and/or analyzed during the current study are available from the corresponding author on reasonable request.

## Abbreviations

BMBF: German Ministry of Education and Research
CDSS: Clinical Decision Support System
COREQ: Consolidated Criteria for Reporting Qualitative Research
DIC: Data Integration Center
DISERDIS: Diagnosis Support in Rare Diseases
EHR: Electronic Health Record
MIRACUM: Medical Informatics in Research and Medicine
OMIM: Online Mendelian Inheritance in Man
RD: Rare Diseases
RDC: Rare Diseases Center
UCD: User-Centered Design Process
WHO: World Health Organization
