# Supplementary material for: Interviews with experts in rare diseases for the development of clinical decision support system software - a qualitative study
Source: BMC Med Inform Decis Mak. 2020 Sep 16;20:230. doi: 10.1186/s12911-020-01254-3 (PMC7493382; doi:10.1186/s12911-020-01254-3)
Supplement: Supplementary file 2 — Additional file 2. Interview guide. [file 12911_2020_1254_MOESM2_ESM.pdf]

# Interview guide for the qualitative study

| Leading question | Control questions | Questions on maintaining the flow of conversation | Notes |
|---|---|---|---|
| **What is the typical diagnostic procedure in the center?** | • Which steps are performed before patient consultation at the center?<br>• What steps are performed after a patient consultation at the center?<br>• Which persons are in contact with the patient in the diagnosis process?<br>• What happens after the diagnosis? | I'd like still to know if ...? | |
| **When do you use software to support diagnosis?** | Use of software tools for diagnosis support | Can you explain this with a concrete situation? | |
| **Who in your center could use a diagnosis support system?** | Users of a diagnosis support system | Can you explain why other users are out of question? | |
| **Which clinical characteristics are important for the diagnosis of rare diseases in your experience?** | Characteristics of Rare Diseases | Can you describe this in more detail?<br><br>For instance …?<br><br>Can you explain this in more detail with an example of a patient case? | |
| **Which clinical characteristics are important for diagnosis of rare diseases in your specialized field?** | Characteristics of certain groups of Rare Diseases<br><br>Which clinical characteristics do certain groups have in common? | Can you explain this in more detail using an example disease? | |
| **Which clinical findings are important for the diagnosis?** | Use of clinical findings: Which clinical findings are particularly relevant? Which are unnecessary? | Can you give some examples? | |

| | | | |
|---|---|---|---|
| **What information do you document about a patient case?** | Documentation of information on a patient (e.g. suspicious diagnoses or letter of physician) | Can you describe this in more detail?<br><br>What do you mean, exactly? | |
| **When do you document findings about patient cases?** | Time of documentation (e.g. during or after the presentation in the center) | | |
| **We are now at the end of the interview. Is there anything else we haven't talked about yet that is relevant for the topic?** | Thanks to the interview partner<br><br>Outlook of the project | | |
