# Supplementary material for: Interviews with experts in rare diseases for the development of clinical decision support system software - a qualitative study
Source: BMC Med Inform Decis Mak. 2020 Sep 16;20:230. doi: 10.1186/s12911-020-01254-3 (PMC7493382; doi:10.1186/s12911-020-01254-3)
Supplement: Supplementary file 3 — Additional file 3. Category system for content analysis. [file 12911_2020_1254_MOESM3_ESM.pdf]

Part A - Initial version of the category system

**Main category**	**Sub category**
Category 1: Diagnostic process	Category 1.1: Steps before a consultation with a patient
	Category 1.2: Steps after a consultation with a patient
	Category 1.3: Persons involved in the diagnostic process
	Category 1.4: Steps after the completion of the diagnosis
Category 2: Use of software tools for diagnostic support	Category 2.1: Users of a clinical decision support system
Category 3: Clinical characteristics of RDs	Category 3.1: Characteristics of groups of RDs
Category 4: Clinical findings	Category 4.1: Usage of findings
	Category 4.2: Relevance of findings
Category 5: Patient documentation	Category 5.1: Time of documentation

Part B - Final version of the category system

The category 1.2 “Steps after a consultation with a patient" was removed. The category 3 “Clinical characteristics of RD" and category 4 “Clinical Findings” were merged, because of overlapping meaning. For the same reason, category 5 “Patient documentation” and sub-category 5.1 "Time of documentation" were collapsed as new category 4 “Patient documentation”.

Main category	Sub category
Category 1: Diagnostic process	Category 1.1: Steps before a consultation with a patient
	Category 1.2: Persons involved in the diagnostic process
Category 2: Use of software tools for diagnostic support	Category 2.1: Users of a clinical decision support system
Category 3: Clinical characteristics and findings of RDs	Category 3.1: Usage of clinical findings
	Category 3.2: Relevance of clinical findings
Category 4: Patient documentation	

Part C – Description of the coding tree

Category	Description
Category 1: Diagnostic process	Description of the diagnostic process from the first contact of a patient up to the diagnosis and treatment.
Category 1.1: Steps before a consultation with a patient	Which steps are performed before a patient is admitted at a center?
Category 1.2: Persons involved in the diagnostic process	Who gets in contact with a patient in the diagnostic process?
Category 2: Use of software tools for diagnostic support	Usage of software tools for diagnostic support in the centers.
Category 2.1: Users of a clinical decision support system	Who could use the CDSS in the diagnostic process?
Category 3: Clinical characteristics and findings of RDs	Which clinical characteristics and findings do rare diseases patients have in common?
Category 3.1: Usage of clinical findings	Who reads and uses the clinical findings?
Category 3.2: Relevance of clinical findings	Which clinical findings are particularly relevant for the diagnosis of rare diseases?
Category 4: Patient documentation	Documentation of information on rare disease patients.
