# Supplementary material for: Interviews with experts in rare diseases for the development of clinical decision support system software - a qualitative study
Source: BMC Med Inform Decis Mak. 2020 Sep 16;20:230. doi: 10.1186/s12911-020-01254-3 (PMC7493382; doi:10.1186/s12911-020-01254-3)
Supplement: Supplementary file 4 — Additional file 4. Example quotations from the interviews. [file 12911_2020_1254_MOESM4_ESM.pdf]

Example quotations from the interviews

Note: Irrelevant sections within comments have not been translated, and are replaced by […].

Quotation number	Quotation (translated into English)	Quotation (original German)	Participant Number
1	“The typical diagnostic route actually operates through our guides and coordinators, where the patients first report at the center and the center guides review the documents […]. And then the patients are referred directly to a B-center, where the patients are then seen or actually first examined by the center guide.”	„Der typische Diagnoseweg läuft tatsächlich über unsere Lotsen und Koordinatoren, wo die Patienten sich eben erstmal bei uns am Zentrum melden und die Lotsen die Unterlagen sichten. Dann geht’s, im Prinzip ist der erste Entscheidungstrack zu sagen, „Okay das geht eindeutig in neurologische Richtung oder eindeutig in eine psychiatrische oder irgendeine andere Richtung“. Und dann den direkten Verweis des Patienten auf ein B-Zentrum, wo die Patienten dann gesehen werden oder tatsächlich auch erstmal Sichtung durch die Lotsen.“	P3
2	“Then there is the third group, where the doctor contacts us and the family doctor and explains that he has been trying for a long time with specialists from different disciplines to clarify this and that he has not found a solution, but he suspects that there might be a rare disease.”	„Dann gibt es die dritte Gruppe, wo der Doktor mit uns Kontakt aufnimmt und der Hausarzt und erklärt, das er seit längerer Zeit mit den unterschiedlichsten Fach-disziplinen versucht das abzuklären und das aber keine Lösung gebracht hat, aber er den Verdacht hat es könnte hier eine seltene Krankheit vorliegen.“	P1

Quotation number	Quotation (translated into English)	Quotation (original German)	Participant Number
3	“Does the patient come on his own initiative, meaning he reports himself, or is he referred to us by another doctor or even another university clinic.”	„Kommt der Patient über eine Eigenzuweisung, also meldet er sich selbst, oder wird er von einem Arzt oder gar einer anderen Uni Klinik an uns überwiesen.“	P7
4	“Then this has an effect on our prioritization, which we make clear. We prioritize submissions from doctors or even submissions from other university hospitals over the patient's own submissions.”	„Dann hat das Auswirkung bei uns auf die Priorisierung, was wir deutlich machen. Wir priorisieren Arzt-Zusendungen oder gar Zusendungen von anderen Universitätskliniken gegenüber den Eigenzusendungen des Patienten.“	P7
5	“What costs us a lot of time is to sift through these findings, assess their value and then come to a meaningful recommendation for the patient.”	„Und dass, was uns dann viel Zeit kostet, ist diese Befunde zu sichten, deren Wertigkeit einzuschätzen und dann für den Patienten zu einer sinnvollen Empfehlung zu kommen.“	P5
6	“Whether we then discuss this in our board with several experts at the same time, or whether it is clear that the patient can be assigned directly to an expert center, e.g. a B-center.”	„Ob wir das dann in unserem Board besprechen mit mehreren Experten gleichzeitig oder ob es dann eben so eindeutig ist, dass man es direkt einem Expertenzentrum also B-Zentrum zuweisen kann.“	P4
7	“The typical diagnosis path actually runs through our center guides and coordinators.”	„Der typische Diagnoseweg läuft tatsächlich über unsere Lotsen und Koordinatoren.“	P3

Quotation number	Quotation (translated into English)	Quotation (original German)	Participant Number
8	“Once a month we have a case conference where we try to solve these patient cases. Every B-center is involved in this.”	„Dann haben wir einmal im Monat eben eine Fallbesprechung, in der wir dann versuchen diese Fälle zu lösen, wenn es uns gelingt. Und da sind dann alle B-Zentren beteiligt.“	P3
9	“There is also the other possibility that my colleague from the non-medical, administrative area is also employed here. She prepares the conversation with the center guide.”	„Also, es gibt auch noch die andere Möglichkeit das unsere, meine Kollegin, aus dem nicht-medizinischen, administrativen Bereich, auch hier eingestellt haben. Und sie bereitet dann das Gespräch mit der Lotsin vor.“	P1
10	“Our team includes laboratory physicians, neurologists, an ENT specialist, a general practitioner, a psychosomatic specialist, a nephrologist, pulmonologists. It’s a motley crew.”	„Wir haben Labormediziner dabei, wir haben einen Neurologen dabei, einen HNO-ler, einen Allgemeinmediziner, einen psychosomatiker, einen Nephrologen, Pulmologen. Also einen bunten Kreis, bunt gemischt.“	P7

Quotation number	Quotation (translated into English)	Quotation (original German)	Participant Number
11	"So at the end of the day we have a framework of an internist with a focus on immunology, which is also the majority of our remaining cases, and we always have a neurologist on board. Apart from that, our center guides rotate through the different B-centers, which brings together different expertise. Currently, the additional doctors (guides) come from pediatrics, because this way developmental disorders are an additional focus. Ultimately, we decide at the case conferences who we involve in the cases again."	„Also letztendlich haben wir halt so ein Grundgerüst aus einem Internisten mit Schwerpunkt Immunologie, was auch Großteil unserer verbleibenden Restfälle ist, und wir haben immer einen Neurologen mit an Board. Und ansonsten wechseln unsere Lotsen durch die verschiedenen B-Zentren durch und dadurch kommen dann auch unterschiedliche Expertisen zusammen. Also derzeit kommen die Lotsen dann, die zusätzlichen Lotsen dann aus der Pädiatrie, weil es da so Entwicklungsstörungen nochmals zusätzlich abgedeckter Schwerpunkt sind. Aber letztendlich entscheiden wir dann auch auf den Fallkonferenzen wen wir in die Fälle dann nochmal mit einbinden."	P5
12	"What I would like to add about our case conferences, is that we usually include a psychosomatic specialist."	„Was ich noch ergänzen möchte zu unserer Fallkonferenz, zu unseren Meetings. Das wir in der Regel auch immer einen Psychosomatiker dabei haben."	P6
13	"Well, it doesn't have to be a B-center. It can also be just a specialist consultation. The B-center actually includes research, teaching and not just diagnosis."	„Naja, es muss nicht unbedingt ein B-Zentrum sein. Das kann auch einfach bloß eine Spezialsprechstunde sein. Zum B-Zentrum gehört ja eigentlich auch Forschung, Lehre und nicht nur die Betreuung."	P1

Quotation number	Quotation (translated into English)	Quotation (original German)	Participant Number
14	"Normally, we process most of the work via the specialist consultations, e.g. via routine care. If it becomes apparent that the gastroenterologist or the ophthalmologist is needed, the RDC is no longer required […]."	„Bei uns ist das in der Regel so, dass wir das meiste über die Facharztsprechstunden, also die Routineversorgung abwickeln. Wenn sich das darstellt, das der Gastroenterologe oder Augenarzt benötigt wird, dann wird das ZSE nicht mehr benötigt und es läuft über die Hochschulsprechstunden."	P1
15	"If the patients still insist on it, then we conduct another case conference in which the most diverse specialists are present."	„Wenn dann die Patienten trotzdem darauf bestehen, dann läuft es so ab, das wir dann nochmal eine Fall-konferenz durchführen, bei der dann die unterschiedlichsten Fachvertreter dabei sind."	I1
16	"But in the end we also decide at the case conferences who we involve in the patient cases. And finally, the heads of the B-centers in particular are a very important source for us."	„Aber letztendlich entscheiden wir dann auch auf den Fallkonferenzen wen wir in die Fälle dann nochmal mit einbinden. Und letztendlich sind insbesondere die Leiter der B-Zentren eine sehr wichtige Quelle für uns".	P5
17	"And if a rare disease is indeed suspected, it will be discussed in an interdisciplinary meeting or case conference and colleagues from other disciplines will be explicitly approached and we will meet."	„Und wenn tatsächlich der Verdacht auf eine Seltene Erkrankung vorliegt, in einem interdisziplinären Meeting oder einer Fallkonferenz besprochen und dabei werden dann explizit Kollegen aus den anderen Fachdisziplinen angesprochen und wir treffen uns dann."	P6

Quotation number	Quotation (translated into English)	Quotation (original German)	Participant Number
18	“And then, there is a big case conference about every two months […], where we discuss special, difficult cases in a kind of seminar, with the goal of obtaining further expert opinions.”	„Und dann gibt es noch etwa alle zwei Monate ein großes Board, das andere nennt sich kleines Board, das öffentlich stattfindet, in der wir dann besondere, schwierige Fälle in einer Art Lehrveranstaltung besprechen, aber auch mit dem Ziel noch Expertenmeinungen zu bekommen.“	P6
19	“Then the patients are referred directly to a B-center, where the patients are then viewed or actually first examined by the center guide.”	„Und dann den direkten Verweis des Patienten auf ein B-Zentrum, wo die Patienten dann gesehen werden oder tatsächlich auch erstmal Sichtung durch die Lotsen.“	P3
20	“And when the center guide has looked through the file, she decides for herself.”	„Und wenn dann die Lotsin die Akte durchgesehen hat, entscheidet sie selbständig.“	P1
21	“We usually have a team of four doctors, each of whom usually takes a case file and works through it to the best of their ability, using the tools I have presented to them.”	„Wir haben ein Team von vier Ärztinnen, sind das in der Regel, von denen nimmt sich jeder in der Regel eine Fallakte und arbeitet die nach bestem Gewissen für sich durch und auch unter anderem unter Verwendung der Tools, die ich ihnen dargestellt habe.“	P7

Quotation number	Quotation (translated into English)	Quotation (original German)	Participant Number
22	“Either we see quite clearly that it is a rare movement disorder and then we would forward the documents very quickly to the appropriate B-center. And it doesn’t cost the center guide most of their time, rather the cases with a bundle of symptoms take a lot of time, which also have been clarified intensively.”	„Entweder wir sehen ganz klar, das ist eine seltene Bewegungsstörung und dann würden wir die Unterlagen sehr schnell in das entsprechende B-Zentrum weitergeben. Und es kostet den Lotsen dann auch nicht den Großteil der Zeit, sondern die Fälle, die uns wirklich viel Zeit kosten, ist so ein Blumenstrauß an Symptomen, der dann häufig auch sehr intensiv abgeklärt wurde.“	P5
23	“The procedure here is, that we can’t examine all of the patients, the outpatient department can’t manage it anymore. […] But the general practitioners just send the results back here, they are looked at. The colleague takes a look at it and says that the findings actually exclude an immune deficiency. That’s exactly the functionality and is what the specialist is doing in the outpatient clinic.”	„Also hier läuft es schon so, dass gar nicht mehr alle Patienten gesehen werden, das schafft die Ambulanz gar nicht mehr. Ich rede jetzt von der Erwachsenenambulanz, weil ich mich da besser auskenne. Aber es schicken die Hausärzte die Befunde einfach hier her, die werden angeguckt. Und der Kollege schaut sich das an und sagt der Befund schließt es eigentlich aus, dass es ein CVID ist. Genau, und das ist eben genau diese Funktionalität, das macht jetzt der Facharzt hier in der Ambulanz.“	P2
24	“Many of the characteristics of patients with rare diseases are common. It’s not that they all have rare symptoms, but they all have very common complaints.”	„Viele der Charakteristika von Patienten mit Seltenen Erkrankungen sind ja alltäglich. Das ist dann nicht so, dass sie alle seltene Symptome haben, sondern	P4

Quotation number	Quotation (translated into English)	Quotation (original German)	Participant Number
		die haben ganz häufige Beschwerden.“	
25	“It is certainly not possible to give generally valid symptoms, because any symptom that is present in a rare disease can also sometimes occur in a common disease. But it always depends on the overall constellation. Basically, I'd say anything that is unusual. When symptoms coincide that are unusual. If the course of disease is unusual and you've just never seen anything like it. Naturally, that makes you think of a rare disease. And also a very early onset and a very unusual course of disease, but I have already said that.”	„Man kann sicherlich keine allgemeingültigen Symptome abgeben, denn jedes Symptom das bei einer Seltenen Erkrankung vorhanden ist, kann auch mal bei einer häufigen Erkrankung auftreten. Sondern es kommt immer auf die Gesamtkonstellation an. Grundsätzlich würde ich sagen, alles was ungewöhnlich ist. Wenn Symptome zusammen aufeinandertreffen, die ungewöhnlich sind. Wenn der Verlauf insgesamt ungewöhnlich ist, sowas hat man einfach noch nie gesehen. Das lässt an eine Seltene Erkrankung natürlich denken. Und dann auch ein sehr früher Beginn und ein sehr ungewöhnlicher Verlauf, ja das habe ich schon gesagt.“	P6
26	“Yes. That depends on the disease group. From the immunological point of view, the antibody constellation ultimately defines the disease together with the clinical symptoms. If we now assume a rare movement disorder, then I’d say it is actually the presentation of the movement disorder	„Ja. Das hängt von der Erkrankungsgruppe ab. Wenn man jetzt vom immunlogischen ausgeht, dann definiert da ja die Antikörper Konstellation letztendlich die Erkrankung gepaart mit passenden klinischen Symptomen dazu. Wenn wir jetzt von einer Seltenen Bewegungsstörung ausgehen, dann würde ich sagen ist, ist es tatsächlich die Präsentation der	P5

Quotation number	Quotation (translated into English)	Quotation (original German)	Participant Number
	coupled with performed genetics. In rare diseases I think it's difficult to find a universal strategy for all these diseases.”	Bewegungsstörung gepaart mit durchgeführter Genetik. In den Seltenen Erkrankungen irgendwie schwierig für all diese Erkrankungen eine allgemeingültige Strategie zu finden.“	
27	“What are we looking at? For us, the neurological findings are the most important thing for now. And then on the same level, the psychopathological findings. That's the first thing you look at. The “clinic“ of symptoms. What I always notice is that the imaging methods, more precisely the morphology of the patients, are very important for pediatricians and human geneticists. Because they are often looking for dysmorphia like head-to-body-ratio and things like that. So first of all basically neurological and psychopathological findings and then also morphology.”	„Was schauen wir uns an? Für uns ist erstmal der neurologische Befund erstmal das Wichtigste. Und dann auf der gleichen Ebene der psychopathologische Befund. Das ist schon so, was man sich als Erstes anschaut. Die „Klinik“ an Symptomen. Und das, was ich immer merke, dass immer für die Kinderärzte und Humangenetiker das Bild, also die Morphologie der Patienten ganz wichtig ist. Weil die eben ganz häufig nach Dysmorphien suchen wie Verhältnis Kopf zu Körper und solche Sachen halt gucken. Also erstmal grundsätzlich neurologischer und psychopathologischer Befund und dann eben auch noch Morphologie.“	P3
28	“Ultimately, many things cannot be depicted in this way, many things are more or less coincidental.”	„Letztendlich, vieles kann man nicht so abbilden, vieles ist mehr oder weniger Zufall.“	P1

Quotation number	Quotation (translated into English)	Quotation (original German)	Participant Number
29	“Well, the family tree definitely is.”	„Also der Familienstammbau auf jeden Fall.“	P1
30	“Phenotype is ultimately the description of the totality of the symptomatology or the visible symptomatology. And if there is an irregular interocular distance, I know that this area was disturbed, e.g. during the growth of the skull. Then certain diseases are more probable, which are associated with the narrow interocular distance. And if you now combine the narrow interocular distance with the kidney, urine or something, you have characterized the phenotype much more closely.”	„Phänotyp letztendlich die Beschreibung der Gesamtheit der Symptomatik oder der sichtbaren Symptomatik. Und wenn ich jetzt einen irregulären Augenabstand habe, dann weiß ich, dass beispielsweise beim Wachstums des Schädels dieser Bereich gestört war. Und dann, kommt dann, dann sind bestimmte Erkrankungen wahrscheinlicher, die eben mit dem engen Augenabstand einhergehen. Und wenn man jetzt den engen Augenabstand in Kombination mit der Niere, Urin oder so, in Kombination bringt, dann hat man schon viel näher den Phänotyp beschrieben.“	P1
31	“They are also phenotypically very different. Many of these diseases involve infections. But there are also plenty that do not have infections and are conspicuous due to autoimmune reactions.”	„Die sind auch phänotypisch sehr unterschiedlich. Was viele dieser Erkrankungen haben, sind Infektionen. Aber es gibt auch viele die haben keine Infektionen und die fallen dann durch Autoimmunreaktionen auf.“	P2
32	“The phenotype. This is a little bit more specific and different with rare diseases that go in the direction of the	Dem Phänotyp. Das ist so ein bisschen spezieller und anders bei den Seltenen Erkrankungen, die in Richtung Psyche gehen. Da ist	P5

Quotation number	Quotation (translated into English)	Quotation (original German)	Participant Number
	psyche. The phenotype-gene correlation is often not as clear and unambiguous as in internal monogenic diseases or in Alzheimer's disease.<br>So, we have the case with the 22.11q deletion syndrome. We have a family, the father has a depression; the indicator patient, the son, has ADHD and a depression. The younger sister, the father's daughter, has a schizophrenic disorder.<br>What I mean to say is that the variability, the phenotypic variability […], is significantly larger than in the somatic field. […]."	einfach diese Phänotyp-Gen Korrelation ganz häufig nicht so klar und eindeutig wie bei den internistischen monogenen Erkrankungen oder bei den Alzheimer Erkrankungen. Also wir haben eben den Fall mit diesen 22.11q Deletionssyndrom. Da ist es, dass wir eine Familie haben, der Vater hat eine Depression, der Indikator Patient der Sohn eine ADHS und eine Depression. Die jüngere Schwester, die Tochter dieses Vaters hat eine Schizophrene Erkrankung. Will sagen, die Variabilität, die phänotypische Variabilität, da will ich mich jetzt deutlich aus dem Fenster lehnen ist deutlich größer als im somatischen Bereich. Deswegen ist es wichtig aber ich würde nicht sagen wie zuverlässig, also bis lang zumindest."	
33	"What is very important for us is that we ask every patient to describe their own medical history and symptoms on 1-2 pages. We ask every patient to do that."	„Was für uns sehr wichtig ist, was wir jeden Patienten auch bitten, wir bitten jeden Patienten auf 1-2 Seiten seine eigene Krankengeschichte und Symptomatik zu beschreiben."	P7
34	"We're talking about 8.000 diseases […]. In the MetabERN, for example, there are 800 different defined diseases which are	„Das hängt wirklich. Wir reden ja über 8000 Krankheiten und wenn wir jetzt die ERNs nehmen, also die Referenznetzwerke. Die haben sich in 24 ERNS unterteilt sozusagen.	P1

Quotation number	Quotation (translated into English)	Quotation (original German)	Participant Number
	all metabolic diseases. Some of them lead to the death of the affected person within a few hours or days if the diagnosis is not made, and for others having a metabolic disease is just an explanation for why the affected person has a disturbed brain development or muscle development or something like that. The findings I need for this are completely different. For one of them I need a lactate value. And for another, I'm not interested in the lactate value at all.”	Ich bin da in mehreren auch mit organisiert. Zum Beispiel in dem MetabERN gibt es 800 verschiedene definierte Krankheiten, die alle Stoffwechselerkrankungen sind. Die einen führen dann dazu, dass so ein Betroffener innerhalb von wenigen Stunden oder Tagen, wenn man die Diagnose nicht stellt, zu Tode kommt und bei dem Anderen ist die Stoffwechselerkrankung lediglich eine Erklärung warum derjenige eine gestörte Hirnentwicklung, Muskelentwicklung oder so etwas hat. Die Befunde, die ich dafür brauche, sind völlig unterschiedlich. Bei dem einen brauche ich einen Lactat Wert. Und bei dem Anderen interessiert mich der Lactatwert überhaupt nicht.“	
35	“As an internist, one naturally always likes to look at the laboratory values. Are there any abnormalities in the laboratory or any abnormalities in imaging? What symptoms does the patient report? These are the central pre-diagnostics. Genetic diagnostics is of course exciting, insofar as it is available. It is often very	„Als Internist schaut man sich natürlich immer gerne die Laborwerte an. Gibt es im Labor Auffälligkeiten, gibt es in der Bildgebung Auffälligkeiten? Was berichtet der Patient für Symptome? Das sind die zentralen Primärdiagnostika. Eine Gendiagnostik ist natürlich spannend, insofern vorhanden. Das ist natürlich häufig sehr komplex muss, ist das so, das lässt sich, nur	P4

Quotation number	Quotation (translated into English)	Quotation (original German)	Participant Number
	complex, of course and only a small minority of our patients bring along a pre-diagnostic genetic test. This is a real rarity.”	eine kleine Minderheit von unseren Patienten, die irgendwie eine vordiagnostische Genuntersuchung mitbringen. Das ist eine wirkliche Rarität.“	
36	“I believe the most important thing for us to get diagnoses is the classical epidemiology of the patient, for instance age, sex and origin. And we also ask for travel anamnesis if you think of rare infectious diseases, for example. We always keep that in mind. And the symptomatology is certainly also important. These are the essential factors that I would consider with patients.”	„Ich glaube das wichtigste für uns, um an Diagnosen zu kommen, ist natürlich ganz klar die klassische Epidemiologie des Patienten, d.h. Alter, Geschlecht und Herkunft. Und das Thema, das bei uns auch immer noch gefordert ist, ist die Reiseanamnese, wenn man z.B. an seltene Infektionskrankheiten usw. denkt. Da denken wir immer noch dran. Und dann natürlich die Symptomatik, ist auch wichtig. Das sind so die wesentlichen Faktoren, die ich bedenken würde bei Patienten.“	P7
37	“We summarize. That’s what the center guides do. They compile a summary letter of what they saw there.”	„Wir fassen zusammen. Das machen die Lotsen dann schon, dass sie eben einen zusammenfassenden Brief Erstellen von dem was sie dort gesehen haben.“	P3
38	“[…] we send the patients a doctor’s letter. The letter tries to represent the essential findings structured […] in terms of comprehensibility and arranged according to	„Also jetzt diese Lotsensachen laufen jetzt endlich so, dass wir Patienten dann einen Arztbrief schicken. Und dass der Arztbrief versucht, die wesentlichen Befunde, einigermaßen strukturiert, aber jetzt nicht strukturiert im Sinne	P5

Quotation number	Quotation (translated into English)	Quotation (original German)	Participant Number
	symptom groups. Then the letter is sent back.”	wie sie jetzt denken von Datenstrukturen. Strukturiert, im Sinne von Verständlichkeit und nach Symptomgruppen geordnet und dann zurückzuschicken.“	
39	“Then there is also the possibility to generate doctor’s letters. That’s what’s available at the end, a summary letter.”	„Und dann gibt es eben auch die Möglichkeit auch Arztbriefe zu erzeugen. Das ist ja quasi das, was am Ende steht, also quasi ein Arztbrief.“	P2
40	“Basically, a summary of the findings is created by a physician, in such a conference we can’t examine 200 pages. But every physician is submitted a summary and this has proven to be very workable.”	„Es wird grundsätzlich eine Zusammenfassung durch einen Arzt der Befunde gemacht, in so einer Konferenz da können wir gar nicht 200 Seiten durchsehen. Aber diese Vignetten dann, die bekommt jeder Arzt dann vorgelegt, und das hat sich als sehr praktikabel erwiesen“.	P6
41	“The result of this case conference, the protocol, is transmitted as a summary letter or diagnostic recommendation to the patient's family doctor or the referring doctor, but not directly to the patient. It’s typically created by Microsoft Word, standard letter.”	„Und dann dieses Ergebnis dieser Fallkonferenz, dieses Protokoll, wird dann sozusagen als Arztbrief oder diagnostische Empfehlung an den Hausarzt des Patienten oder den zuweisenden Arzt übermittelt, nicht an den Patienten direkt. Der wird erstellt, per Word. Klassisch per Word, Standardbrief.“	P7
42	“We document our findings, on a standardized questionnaire. Or even the	„Wir dokumentieren dann, faktisch auch auf einem standardisierten Fragebogen, dokumentieren wir	P1

Quotation number	Quotation (translated into English)	Quotation (original German)	Participant Number
	result of the case conference”.	dann eben unsere Befunde, die dann gemacht wurden. Oder eben auch das Ergebnis der Fallkonferenz.“	
43	“If, for example, exome sequencing is indicated, it will also be documented.”	„Wenn z.B. eine Exom-Analyse indiziert ist, dann wird das eben auch dokumentiert.“	P6
44	“Yes, there is an extra form for this in our EHR system. […] We document these case conferences. In our center, we not only code each patient with regard to a rare disease with an Orphanet code, but we also document these interdisciplinary case conferences in a separate form.”	„Ja, dafür gibt es in unserem KIS-System ein extra Formular, wir haben ORBIS, erstellt worden. Wir haben hier die Besonderheit in x, das wir nicht nur jeden Patienten hinsichtlich einer Seltenen Erkrankung kodieren, mit einem Orphanet-Code, sondern das wir dann eben auch diese Fall-konferenzen, diese interdisziplinären Fall-konferenzen dokumentieren, in einem eigenen Formular.“	P6
45	“This is done throughout the entire process, not just at the end. At the beginning, this means that the form also contains the necessary entry, is there only a suspicion or is the diagnosis certain. And you can follow along the patient history if something has changed, with every new stay of a patient. Because it is possible that a patient	„Das wird eben schon im gesamten Prozess gemacht, nicht erst zum Abschluss. Das heißt am Anfang, das Formular beinhaltet auch den notwendigen Eintrag, besteht nur ein Verdacht oder ist die Diagnose sicher. Und man kann sozusagen, entlang der Patientenhistorie, bei jedem neuen Aufenthalt eines Patienten, dann verfolgen, hat sich da was geändert. Denn es ist ja auch mal möglich, dass ein Patient erstmal nur eine bestimmte	P6

Quotation number	Quotation (translated into English)	Quotation (original German)	Participant Number
	initially only gets a certain suspected diagnosis „A", but then in the course of time a suspected diagnosis "B" is more likely to be accepted, which may then be confirmed. And that's what you can follow along a patient's history very clearly".	Verdachtsdiagnose „A" bekommt, dann aber im Verlauf der Zeit doch eher eine Verdachtsdiagnose „B" angenommen wird, und die sich dann möglicherweise auch bestätigt hat. Und dies lässt sich entlang der Historie des Patienten ganz klar verfolgen."	
46	"In theory, we have an EHR system. Our patients are not registered in the EHR. Our patients are, so to speak, registered in a separate database. It's a proprietary database tool."	„Theoretisch haben wir ein ORBIS, wie Sie. Agfa ORBIS als KIS. Unsere Patienten sind nicht im KIS erfasst. Unsere Patienten sind sozusagen in einer eigenen Datenbank erfasst. Das ist ein eigenes Datenbanktool."	P7
47	"We also have a system for our administrative patient admission. We enter the patient's master data there."	„Was wir noch haben ist ein System für unsere administrative Patientenaufnahme. Was wir da machen, da erfassen wir die Stammdaten des Patienten."	P7
48	"Medication therapy is a relatively advanced documentation tool, which also indicates [pharmacological] interactions etc., even during documentation. This also directly indicates when the combination of these drugs is perhaps not so good. Exactly, there are a	„Medikation Therapie, ist hier ein relativ fortschrittliches Dokumentationstool, was auch auf Wechselwirkungen usw. hinweist, also auch während der Dokumentation. Da bekommt man auch direkt angezeigt, dass die Kombination zwischen diesen Medikamenten vielleicht nicht so gut ist (..). Genau, da gibt es ein paar Spezialfelder für die	P2

Quotation number	Quotation (translated into English)	Quotation (original German)	Participant Number
	few special fields for immunology, namely immunoglobulin therapy, etc. Basically, a list is built up, so one can document the entire history of immunoglobulin therapy.”	Immunologie, nämlich die Therapie der Immunglobuline usw. Da baut sich im Prinzip so eine Liste auf, also im Prinzip kann man da, die gesamte Historie der Immunglobulinen-Therapie dokumentieren.“	
49	“The most important is the diagnosis list, which is also in the ICD-10. Then there are different catalogs which you can browse. These are immune deficiency diagnoses, for example. So if you now have a patient with an immune deficiency, you could enter it here. One can always enter free text. Concerning genetics, there should now also be a list of genes.”	„Das wichtigste ist die Diagnoseliste, die auch im ICD-10 ist. Dann gibt es hier verschiedene Kataloge in denen man drin suchen kann. Das sind jetzt zum Beispiel PID-Diagnosen. Also wenn man jetzt einen Patienten hat mit CVID, da könnte man das hier so eintragen. Freitext darf man auch immer hinterlegen, Genetik, da müsste jetzt auch eine Liste von Genen kommen.“	P2
50	“No we currently use the internet as a platform and classic tools such as Medline. But we haven’t set up an IT structure yet.”	„Nein wir verwenden derzeit das Internet als Internetplattform, klassischen Tools wie Medline über Leitlinien. Aber wir haben jetzt keine IT-Struktur aufgebaut.“	P5
51	“Yes, of course. Literature research with common databases like OMIM, Orphanet, Pubmed. These are used, of course. Phenomizer is something which is used in exome	„Ja, natürlich. Literatur-recherchen. Die normalen Databasen wie OMIM, Orphanet, Pubmed. Das wird natürlich benutzt. Der Phenomizer ist etwas das dann im Rahmen der Exom-Diagnostik	P6

Quotation number	Quotation (translated into English)	Quotation (original German)	Participant Number
	diagnostics and we definitely do that.”	genutzt wird und das machen wir auf jeden Fall.“	
52	“We have never used FindZebra. But apart from that we try to use tools, although I cannot tell you exactly what my colleagues use.”	„Dieses „FindZebra“ haben wir noch nie benutzt. Aber ansonsten wird schon versucht Tools zu benutzen, im Einzelnen kann ich ihnen gar nicht sagen was da Frau (..) macht.“	P1
53	“In human genetics, yes. I'm a psychiatrist and neurologist and, to be honest, I haven't used the tools yet. I'm a spokesman for the center. I can’t remember the name of the tool. Can you tell me the names of some of these tools?”	„In der Humangenetik und dort ja. Ich bin ja Psychiater und Neurologe und habe die ehrlich gesagt noch nicht genutzt. Ich bin ja Sprecher von dem Zentrum. Ich weiß gar nicht mehr wie das Tool hieß. Sagen sie mal so ein paar Namen von den Dingern.“	P3
54	“I’m not familiar with it, I believe rather infrequently. Because I have the impression that maybe my colleagues consider themselves to be experts, but maybe I am going too far with this.”	„Ist mir nicht bekannt, ich glaube eher selten. Weil mein Eindruck ist, vielleicht lehne ich mich da zu weit aus dem Fenster, die Kollegen halten sich schon selbst für Experten.“	P2
55	“On the one hand there are the special tools, of course, like FindZebra or Phenomizer, but we also strongly refer to Isabel Healthcare for example,	„Das sind zum einen natürlich die Spezialtools wie FindZebra oder auch den Phenomizer, aber wir nehmen unter anderem auch starken Bezug auf Isabel Healthcare zum Beispiel, was ja eher nochmal generalistischer	P7

Quotation number	Quotation (translated into English)	Quotation (original German)	Participant Number
	which is more general. We can also access ADA.”	aufgestellt ist. Wir haben auch mit ADA, da können wir auch drauf zugreifen.“	
56	“Occasionally you search the internet, of course. But in my opinion there is nothing really satisfying or much at all when searching for prevalent rare diseases, which you can’t identify for certain. Well, I think there is a need for it.”	„Also gelegentlich sucht man natürlich im Internet, aber es gibt aus meiner Sicht nichts so richtiges befriedigendes oder Vieles, wenn man dann nach gängigen Seltenen Erkrankungen sucht, die man dann auch nicht entsprechend sicher identifizieren kann. Also ich denke schon, dass es da einen Bedarf gibt.“	P4
57	“Who? Definitely the doctors.”	„Wer? Ja auf jeden Fall die Ärzte.“	P6
58	“That varies. I would say at a university center like ours, I think they [the users] are primarily the colleagues, the medical colleagues at the center, but definitely also those at the B-centers.”	„Das ist unterschiedlich. Ich würde sagen an einem Universitätszentrum wie unserem, denke ich sind das primär die Kollegen, die ärztlichen Kollegen am Zentrum, aber durchaus die am B-Zentrum.“	P6
59	“I think it’s the profile of the center guide.”	„Ich denke, also bei uns das Lotsenprofil.“	P7
60	“Well, at the first level definitely the center guide, but at the second level the specialists in the B-centers.”	„Also a) in der ersten Ebene auf jeden Fall der Lotse, aber auf der zweiten Ebene dann die Fachärzte in den B-Zentren.“	P3
61	“It might make the center guide's job easier if it's something groundbreaking, but of course not if it's 20	“Dem Lotsen wird es vielleicht die Arbeit einfacher machen, wenn es irgendwas Richtungsweisendes ist, natürlich nicht, wenn dann 20	P4

Quotation number	Quotation (translated into English)	Quotation (original German)	Participant Number
	differential diagnoses. Then the guide doesn’t get anywhere with it either. But in the end I think it is even more important for the experts in the B-centers. I definitely think that.“	Differenzialdiagnosen aufpoppen. Dann kommt der Lotse damit auch nicht weiter. Aber ich glaub letztlich noch mehr von Bedeutung ist es für die Fachleute in den B-Zentren. Das denke ich schon.“	
62	“Only to a limited extent for the non-physician center guides. They simply lack expertise, it must be said. They can do certain things, e.g. when it comes to selecting codes or something like that, I think that's okay, but for clinical-medical evaluation, that's not what they're trained for.”	„Nur eingeschränkt bei den Nicht-Ärztlichen Lotsen. Den fehlt einfach die Expertise, das muss man ganz klar sagen. Die können bestimmte Dinge, wenn es z.B. darum geht, irgendwelche Kodes oder sowas rauszusuchen, ich denke das ist okay, aber für die klinisch-medizinische Bewertung, dafür sind sie ja nicht ausgebildet.“	P6
63	“I would already place it with the medical center guides who are located at the RDC. It also somewhat depends on the quality of the tool. If I still need additional expert knowledge to be able to interpret what the tool spits out to me, then I'd rather see the expert do it. If the tool is so great that I enter three parameters and it tells me the diagnosis with 99% probability, then of course it is ideal for a center guide. […].”	„Ich würde es schon bei den ärztlichen Lotsen ansiedeln, die sich im ZSE befinden. Das kommt auch etwas auf die Qualität des Tools an. Wenn ich noch zusätzliches Expertenwissen brauche, um das was mir das Tool ausspuckt, interpretieren zu können. Dann würde ich es eher bei dem Experten angesiedelt sehen. Wenn das Tool so super ist, dass ich drei Parameter eingebe, und dann sagt es mir mit 99%iger Wahrscheinlichkeit die Diagnose, dann ist es natürlich ideal für einen Lotsen. Ist aber auch die Frage, wie	P2

Quotation number	Quotation (translated into English)	Quotation (original German)	Participant Number
		oft ist es falsch negativ. Wird also quasi verhindert, dass sich das Experte nochmal anschaut und sagt, das ist keine primäre Immundefizienz, sondern eben was anderes.“	
64	“When I think it might be a rare disease, and I send the patient to the center guide, then it's helpful for the center guide because he may not know what kind of rare disease it is. So from my point of view it would be even more important to start earlier. So either with someone who is not at the center for Rare Diseases at all, or is somewhere in the clinic. It would be very helpful there.”	„Wenn ich dran denke, es könnte eine Seltene Erkrankung sein und schick den dann zu einem Lotsen. Dann ist es natürlich hilfreich für den Lotsen, weil er vielleicht nicht weiß, was für eine Seltene Erkrankung das ist, da zu unterstützen. Also aus meiner Sicht noch wichtiger wäre es früher schon anzusetzen. Also entweder bei jemand der gar nicht im Zentrum für Seltene Erkrankungen ist, oder irgendwo in der Klinik ist. Da wäre es schon sehr hilfreich, und wenn man es noch weiterspinnt.“	P2
65	“I believe there are two groups. If I’ve understood your system correctly, you need someone to do the data entry. After all, data entry usually has to be structured, at least standardized, in a certain way. I would see the medical user as being limited there. He doesn't have a feeling for why this	„Ich glaube es gibt zwei Gruppen. Wenn ich ihr System verstanden habe, benötigt man erstmal jemand, der die Dateneingabe macht. Denn die Dateneingabe, muss ja in der Regel strukturiert erfolgen, zumindest standardisiert, in einer bestimmten Art und Weise. Da würde ich den ärztlichen Anwender eher nur bedingt sehen. Der hat da nicht das Gespür für warum überhaupt und warum das wichtig	P7

Quotation number	Quotation (translated into English)	Quotation (original German)	Participant Number
	might be important. Because it requires a lot of work. For me, this would be more in the area of a coding specialist or similar, concerning the requirement profile. You can place him [medical doctor] in the search field, especially if he has to evaluate whether the cases have a certain similarity.”	wäre. Weil das schon einen hohen Arbeitsaufwand erfordert. Das würde für mich eher in den Bereich Kodierfachkraft oder ähnliches, vom Anforderungsprofil zutreffen. Dann im Bereich der Suche kann man ihn schon einsetzen im Bereich der Suche vor allem, wenn er beurteilen sollen, haben die Fälle eine gewisse Ähnlichkeit.“	
66	“In our case discussions, everyone has a tablet computer in front of them. And everyone has access to these diagnostic support systems and knows them. Usually we use them together on the beamer. Then somebody goes, e.g. to FindZebra, and enters symptoms. And then we discuss the results. Our doctors have licenses for those systems. Of course you have to say, they are only used by the doctors.”	„Also bei uns in der Fallkonferenz hat eigentlich jeder ein Tablet vor sich. Und jeder sozusagen hat auch Zugriff auf diese Systeme und kennt die. Meistens nutzen wir die sozusagen gemeinsam am Beamer sozusagen. Wir dann gesagt: ja geh doch mal in FindZebra und gib doch mal ein. Und dann über diese Liste die dann kommt wird aktiv diskutiert. Unsere Mitarbeiter und die freiwilligen Mitarbeiter sozusagen, die haben auch Lizenzen für Isabel jetzt, für FindZebra, die kennen die und können die auch so nutzen. Das sind natürlich muss man sagen, die werden nur durch die ärztlichen Mitarbeiter genutzt.”	P8
